# *Pleurotus tuber-regium* Polysaccharides Attenuate Hyperglycemia and Oxidative Stress in Experimental Diabetic Rats

**DOI:** 10.1155/2012/856381

**Published:** 2012-08-30

**Authors:** Hui-Yu Huang, Mallikarjuna Korivi, Ying-Ying Chaing, Ting-Yi Chien, Ying-Chieh Tsai

**Affiliations:** ^1^Department of Food Science, Nutrition, and Nutraceutical Biotechnology, Shih Chien University, Taipei City 10462, Taiwan; ^2^Division of Mental Health and Addition Medicine, Institute of Population Health Sciences, National Health Research Institutes, Zhunan 35053, Taiwan; ^3^Laboratory of Exercise Biochemistry, Department of Sports Sciences, Taipei Physical Education College, Taipei City 11153, Taiwan; ^4^Institute of Biochemistry and Molecular Biology, National Yang-Ming University, Taipei City 11221, Taiwan

## Abstract

*Pleurotus tuber-regium* contains polysaccharides that are responsible for pharmacological actions, and medicinal effects of these polysaccharides have not yet been studied in diabetic rats. We examined the antidiabetic, antihyperlipidemic, and antioxidant properties of *P. tuber-regium* polysaccharides in experimental diabetic rats. Forty rats were equally assigned as diabetic high-fat (DHF) diet and polysaccharides treated DHF groups (DHF+1P, DHF+2P, and DHF+3P, 20 mg/kg bodyweight/8-week). Diabetes was induced by chronic low-dose streptozotocin injections and a high-fat diet to mimic type 2 diabetes. Polysaccharides (1P, 2P, and 3P) were extracted from three different strains of *P. tuber-regium*. Fasting blood glucose and glycosylated hemoglobin (HbA1c) levels substantially decreased, while serum insulin levels were restored by polysaccharides treatment compared to DHF. Furthermore, plasma total cholesterol, triglycerides, and low-density lipoprotein levels were significantly (*P* < 0.01) lower in polysaccharide groups. High-density lipoprotein levels were attenuated with polysaccharides against diabetes condition. Polysaccharides inhibited (*P* < 0.01) the lipid peroxidation index (malondialdehyde), and restored superoxide dismutase and glutathione peroxidase activities in the liver of diabetic rats. The antihyperglycemic property of polysaccharides perhaps boosts the antioxidant system that attenuates oxidative stress. We emphasize that *P. tuber-regium* polysaccharides can be considered as an alternative medicine to treat hyperglycemia and oxidative stress in diabetic rats.

## 1. Introduction

Mushrooms and their ingredients are commonly used food substances among East Asians. *Pleurotus tuber-regium*, a popular edible mushroom, has been considered as a profound health promoting mushroom in traditional Chinese medicine [1, 2]. In addition to nutritive values,* P. tuber-regium* also exhibits some medicinal properties, including relief for stomach ailments, fever, asthma, smallpox, high blood pressure, and cancer [3, 4]. The fruit bodies of *P. tuber-regium* are rich in protein, while sclerotium is rich in fiber, especially nonstarch polysaccharides [5], mainly composed of bioactive *β*-glucans responsible for pharmacological actions [6, 7]. 

Due to widespread changes in dietary intake, incidence of obesity and diabetes has been increasing around the world including Asian counties. Diabetes is a cluster metabolic disorder characterized by hyperglycemia resulting from the body's inability to use blood glucose for energy [8, 9]. Hyperglycemia along with hyperlipidemia leads to severe morbidity and death [10, 11]. Furthermore, excessive production of reactive oxygen species (ROS) in diabetic animals causes oxidative stress that plays a major role in diabetes-associated cardiovascular and fatty liver diseases [12, 13]. Under such condition, intake of high-fat diet further worsens the oxidative milieu and compromises the cellular functions. Since antidiabetic drugs produce negative effects on other metabolisms [14], supplementation of antihyperglycemic substances, which also possess antioxidant properties, might be an alternative therapy to overcome this critical condition. 

Antihyperglycemic and antioxidant properties of fresh mushroom species and their polysaccharide extracts have been demonstrated [13, 15]. However, most of the studies have been performed either with fruiting bodies or mycelia of mushroom, not with culture media of *P. tuber-regium*. It is well established that polysaccharides act as effective antidiabetic, antioxidant substances and boost immunity [4, 7, 12]. Since polysaccharides exhibit diverse biological activities, extracellular polysaccharides that are released into the culture medium during submerged fermentation of *P. tuber-regium* are medicinally important. Moreover, these polysaccharides are low cost with significant therapeutic applications. The potential antihyperglycemic and antioxidant properties of polysaccharides of *P. tuber-regium* in diabetes fed a high-fat diet have not been analyzed.

Therefore, this study aimed to examine the antihyperglycemic, antilipidemic, and antioxidant properties of *P. tuber-regium* extracellular polysaccharides under diabetes-induced adverse conditions. In our study, we induced experimental diabetes in rats by chronic low dose of STZ injections and high-fat diet, which mimics the features of type 2 diabetes [16, 17]. In order to evaluate strain specific beneficial effects, we chose three different strains of *P. tuber-regium* and the polysaccharides were extracted from each strain and administered to diabetic rats. 

## 2. Materials and Methods

### 2.1. Extraction of Polysaccharides from Three Different Strains

The cultures strains of *Pleurotus tuber-regium*, including MUCL-39359, MUCL-44597, and MUCL-44822, were obtained from the Mycotheque Catholique de Louvain, Louvain-la-Neuve, Belgium. The mycelia of* P. tuber-regium* were cultured in 300 mL Erlenmeyer flasks containing 100 mL broth for 20 days (6.5% glucose, 0.30% soy peptone, 1% yeast extract, 0.01% MgSO_4_, and 0.02% KH_2_PO_4_ in distilled water at pH 5.5). The culture broth was separated from the mycelia by filtration and then freeze-dried for experimental use. The polysaccharide preparation was performed at the Institute of Biochemistry and Molecular Biology, National Yang-Ming University, Taipei, according to the protocols as described previously [18–20]. The polysaccharides were extracted from the broth using heat. After neutralization of the acidic medium (0.1 mol/L NaOH), followed by the addition of 1% NaCl and precipitated in ethanol (1 : 5 v/v), the precipitate was separated by centrifugation in an ethanol-hydrogen peroxide solution (1 : 1 v/v) and second extraction with ethanol (1 : 4 v/v). The amount of crude extracellular polysaccharides present in each strain was quantified by phenol-sulfuric acid method. The polysaccharides extracts from three different strains, including MUCL-39359, MUCL-44597, and MUCL-44822 were labeled as 1P, 2P, and 3P. The freeze-dried polysaccharides were freshly prepared prior administration to rats by dissolving in required quantity of HPLC grade distilled water.

### 2.2. Animal Care and Maintenance

Eight-week-old male Wistar rats weighing 180 g ± 20 g were obtained from the National Laboratory of Animal Breeding and Research Center, Taipei, Taiwan. The rats were housed at 23 ± 2°C temperature with an alternating 12 h dark and light cycle. All rats were fed a high-fat diet (22%) and water *ad libitum*. The standard diet AIN-93 formula, which composed of 72.6% carbohydrates, 4% fat, and 14% protein, was modified as 54% carbohydrate, 22% fat, and 14% protein in order to produce the high-fat diet. Additional 18% of fat (lard, an animal fat) was added to the fat portion of the standard diet. Changes in bodyweight on every other day and daily food intake were recorded throughout the study. The experimental procedures were conducted according to the ethical guidelines and this study was approved by the Institutional Animal Ethics Committee of Shih Chien University. 

### 2.3. Chemicals

Streptozotocin (STZ) and all other chemicals were obtained from the Sigma Chemical Co., (St. Louis, MO, USA). The kits were indicated under specific sections. 

### 2.4. Induction of Experimental Diabetes

After one week acclimatization to laboratory conditions, rats were fasted for 12 h before an intraperitoneal injection of STZ. The STZ solution was freshly prepared in 0.1 M citrate buffer (pH 4.5), and 10 mg/kg bodyweight (bw) was injected in a volume of 1 mL/kg bw, along with nicotinamide (30 mg/kg bw) for every other day. In order to achieve a rat model with type 2 diabetes, all STZ injected rats were fed on a high-fat diet throughout the study. Neither death nor any other adverse symptoms were observed at the tested concentrations. Blood glucose levels were monitored every three days, and rats with high blood glucose levels (≥200 mg/dL) after 6 weeks were considered as hyperglycemic/diabetic rats.

### 2.5. Grouping and Treatment

Forty rats were equally divided into four groups, including diabetic high-fat diet (DHF), diabetic high-fat diet plus polysaccharides-1P (DHF+1P), diabetic high-fat diet plus polysaccharides-2P (DHF+2P), and diabetic high-fat diet plus polysaccharides-3P (DHF+3P). Each group consisted of 10 rats. Rats in all groups received chronic low dose of STZ injections on every other day as described previously and provided high-fat diet throughout 8-week period. Except DHF group, remaining three groups were orally administered with 1P, 2P, and 3P strains polysaccharides at the dose of 20 mg/kg bodyweight per day for 8-week period. 

Fasting blood samples were collected from the tail vein for every three days and also at week 6 and week 8. All rats were sacrificed under anesthesia (chloral hydrate, 400 mg/kg bw, intraperitoneal) after completion of 8-week treatment and liver was quickly excised, washed with ice cold saline, and adhesive blood was removed and immediately stored in liquid nitrogen for further biochemical analyses. 

### 2.6. Biochemical Analyses

#### 2.6.1. Oral Glucose Tolerance Test (OGTT)

Six weeks after STZ injections and polysaccharide treatment, OGTT was performed for all rats to evaluate their glucose tolerance. All rats were fasted for 8 h and OGTT was conducted between 7.00 am and 9.00 am. All rats were orally administered with 50% glucose solution (w/v, 1 g/kg bw). Blood samples were collected from the tail vein by tail milking at 0, 30. 60, 120, and 180 min time points after glucose administration. Blood glucose values were determined by the glucose analyzer (Lifescan, Milpitas, CA, USA).

#### 2.6.2. Measurement of Blood Glucose, Serum Insulin, and Glycosylated Hemoglobin Levels

Blood glucose levels were estimated after 8-week polysaccharide treatment as described in the previous section. Serum insulin levels were measured by an enzyme-linked immunosorbent assay (ELISA) with an antiinsulin monoclonal antibody. The serum sample was quantified by ELISA analyzer (Tecan Genios, A-5082, Austria) by using commercial ELISA kits (Diagnostic Systems Laboratories, Webster, TX, USA) and followed according to the manufacturer's protocol. 

Glycosylated hemoglobin (HbA1c) concentration was measured in blood samples on the same day after blood collection according to the manufacturer's protocol as described in the kit (Randox, Antrim, UK).

#### 2.6.3. Estimation of Plasma Lipid Profiles

To evaluate the effect of polysaccharides supplementation on chronic STZ plus high-fat diet-induced adverse effect on lipid profiles, plasma total cholesterol (TC), triglycerides (TG), high-density lipoprotein (HDL), and low-density lipoprotein (LDL) levels were estimated spectrophotometrically using Vitros DT60 II analyzer (Johnson and Johnson Clinical Diagnostics Inc., Rochester, New York, USA). All the values are expressed as mg/dL.

#### 2.6.4. Evaluation of Hepatic Lipid Peroxidation Index and Antioxidant Enzymes

Lipid peroxidation marker in the liver homogenate was determined by measuring the malondialdehyde (MDA) levels as described by Ohkawa et al. [21]. The MDA concentration was calculated per mg protein and expressed as nanomoles of MDA per mg protein.

Superoxide dismutase (SOD) and glutathione peroxidase (GPx) activities were assayed in the liver homogenates according to commercial kits (Cayman Chemical Company, USA). For SOD, the absorbance of reaction mixture was read at 450 nm on ELISA plate reader (Tecan Genios, A-5082, Austria), and activity was expressed as units/mg protein. GPx activity was estimated by using NADPH, and the reduction in absorbance was read at 340 nm for every minute to obtain at least 5 time points by using a plate reader (Tecan Genios, A-5082, Austria). Enzyme activity was calculated per mg protein and expressed as nanomoles/mg protein/min. The protein concentrations in liver homogenates were determined according to the Bio-Rad protein assay procedure (Richmond, California, USA).

### 2.7. Statistical Analyses

All the data were expressed as mean ± SEM for ten replicates (10 individual animals in each group). The significant difference among groups was analyzed by using one-way analysis of variance (ANOVA) along with Tukey's multiple-range post-hoc test. The statistical differences were considered significant at *P* < 0.05. The data were analyzed by MS Office Excel and SPSS software. 

## 3. Results

### 3.1. Percentage of Polysaccharides in Three Different Strains of *P. tuber-regium*

Table 1 shows the percentage of polysaccharides that are presented in each strains of *P. tuber-regium*. For the first time, we extracted the polysaccharides from three different strains, and recorded that strain MUCL-39359 (1P) has a higher percentage of polysaccharide (8.18%) than strain MUCL-44597 (6.24%) and strain MUCL-44822 (3.99%).

### 3.2. Influence of Polysaccharides on Food Intake, FCE Ratio, and Bodyweight Changes

The average food intake for all groups was measured daily, and found no significant difference with polysaccharide supplementation compared to DHF group. In addition, food conversion efficiency (FCE) was also not significantly altered among the groups (Table 2).

Initial bodyweights were not significantly different among four groups. However, during the course of study, lower bodyweights (*P* < 0.01) were recorded at week 2, 4, 6, and 8 in all polysaccharides received groups compared to DHF group. Intake of high fat-diet along with STZ injections (DHF group) resulted in a greater increase in whole bodyweight (from 302.12 ± 2 g to 499.8 ± 2.9 g). The overall weight gain in the DHF group was 197.67 ± 4 g over a period of week 8. Among three polysaccharides, DHF+1P, which received high percentage polysaccharides, showed moderately lower weight gain (160 ± 4 g) than DHF+2P (190 ± 3.5 g) and DHF+3P groups (188 ± 3 g) (Table 3).

### 3.3. Polysaccharides Supplementation on Blood Glucose, Insulin and HbA1c Levels

The fasting blood glucose in DHF group estimated after 8 weeks was 368 ± 2.3 mg/dL, which represents the severe diabetic condition. However, oral administration of polysaccharides significantly (*P* < 0.01) decreased blood glucose levels. This reduction was more prominent in 1P polysaccharides than 2P and 3P polysaccharides (Table 4).

Rats received STZ plus high-fat diet for weeks 8 showed lower insulin levels (0.3 *μ*U/mL), which reflects that chronic STZ injection destroyed the pancreatic *β*-cells. Nevertheless, rats supplemented with polysaccharides along with STZ plus high-fat diet showed restored (*P* < 0.01) insulin levels compared to the DHF group. The restored insulin with 1P (0.9 *μ*U/mL) was prominent than 2P and 3P polysaccharides (Table 4).

In addition to the above two diabetic elements, another striking characteristic feature of diabetes was evidenced by higher HbA1c in DHF group. Interestingly, we found significantly (*P* < 0.01) lower HbA1c in diabetic rats treated with polysaccharides. The decreased HbA1c may be associated with percentage of polysaccharides present in each strain, since DHF+1P showed relatively higher inhibition (~40%) than 2P and 3P polysaccharides (Table 4).

### 3.4. Polysaccharides Improved Oral Glucose Tolerance

**Figure 1:**
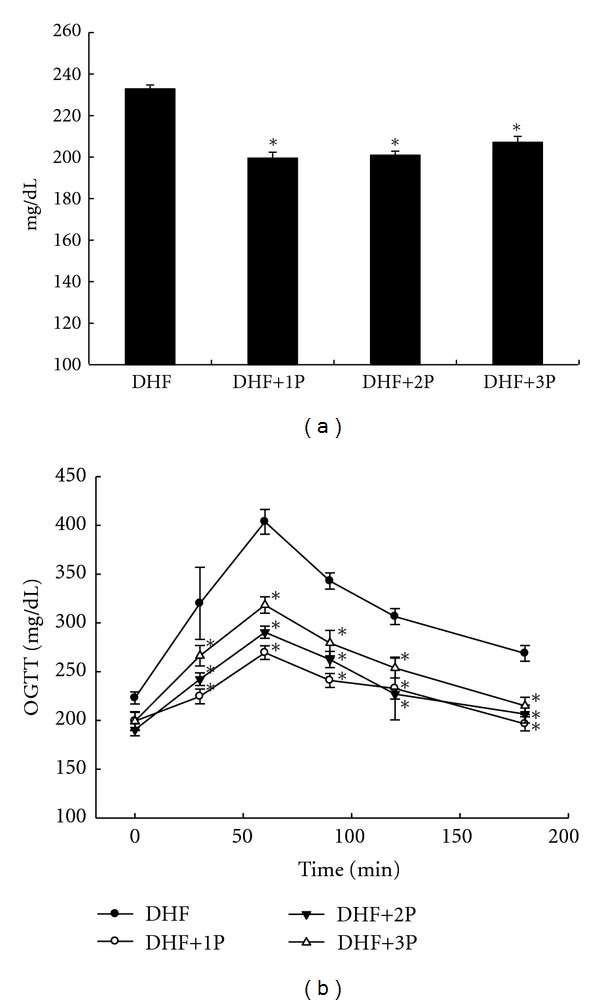
Fasting blood glucose levels (a) and oral glucose tolerance test (b) after 6-week polysaccharides (1P, 2P, and 3P) treatment in STZ-injected diabetic rats with high-fat diet rats (DHF). The ∗ indicates a significant difference compared to DHF group (*P* < 0.01).

Supplementation of polysaccharides along with STZ plus high-fat diet has been shown to improve the oral glucose tolerance compared to untreated (DHF) group at week 6 (Figure 1(a)). This was evidenced by significantly (*P* < 0.05) lower blood glucose values at all time points (except baseline, 0 min) in polysaccharides-treated rats (Figure 1(b)).

### 3.5. Antihyperlipidemic Property of Polysaccharides

The estimated TC, TG, and LDL levels in plasma samples were significantly (*P* < 0.01) lower in all polysaccharides-treated groups compared to DHF group. In this study, decreased TC (23%) and LDL (21%) were more profound in DHF+1P group compared to other two polysaccharides received groups. Moreover, plasma HDL were significantly (*P* < 0.01) increased against diabetic condition in all polysaccharide supplemented groups, and this increase was higher in DHF+1P group (Table 5).

### 3.6. Effect of Polysaccharides against Lipid Peroxidation in the Liver

**Figure 2:**
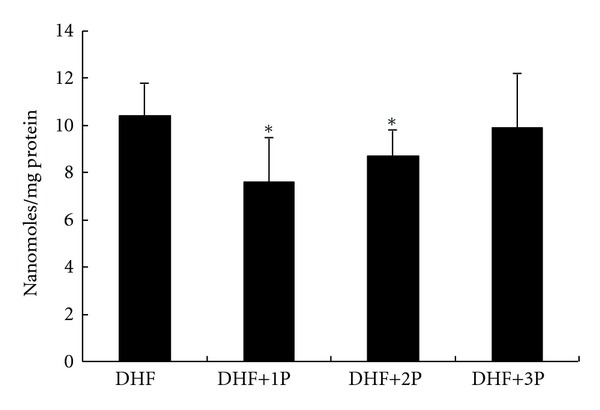
Polysaccharides (1P, 2P, and 3P) impact against lipid peroxidation (malondialdehyde (MDA) levels) in the liver of diabetic rats fed a high-fat diet (DHF). The ∗ indicates a significant difference compared to DHF group (*P* < 0.01).

STZ injections plus high-fat diet-induced lipid peroxidation in liver samples was determined by monitoring the MDA levels. We found MDA levels were significantly (*P* < 0.01) lower in DHF+1P and DHF+2P groups compared to DHF group, which indicates attenuated lipid peroxidation by polysaccharides supplementation. Nevertheless, 3P polysaccharides (DHF+3P) showed no significant change (Figure 2).

### 3.7. Antioxidant Effect of Polysaccharides

**Figure 3:**
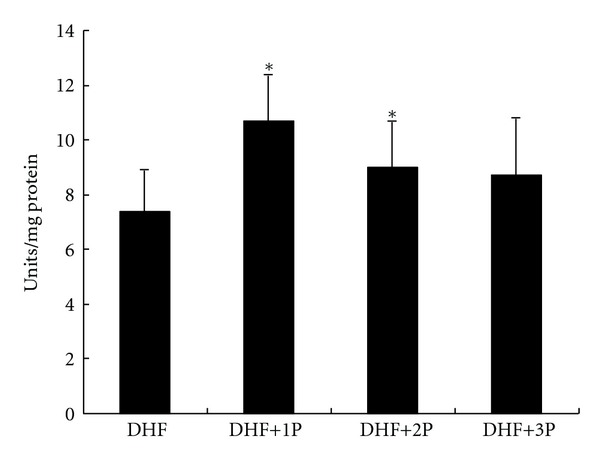
Superoxide dismutase (SOD) activity in the liver of diabetic rats fed a high-fat diet (DHF) and treated with three different polysaccharides (1P, 2P, and 3P). The ∗ indicates a significant difference compared to DHF group (*P* < 0.01).

**Figure 4:**
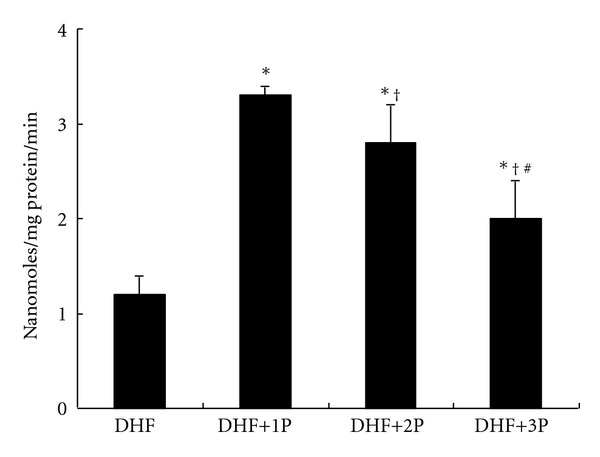
Glutathione peroxidase (GPx) activity in the liver of diabetic rats fed a high-fat diet (DHF) and treated with three different polysaccharides (1P, 2P, and 3P). The ∗ indicates a significant difference compared to DHF group (*P* < 0.01), † indicates a significant difference compared to DHF+1P (*P* < 0.05), and # indicates a significant difference compared to DHF+2P (*P* < 0.05).

SOD activity was significantly (*P* < 0.05) higher in DHF+1P and DHF+2P groups compared to untreated DHF group. Similar to MDA data, 3P polysaccharide, which represents for low-percentage polysaccharide, was unable to restore the SOD activity (Figure 3). Another major antioxidant enzyme, GPx, activity was significantly (*P* < 0.01) elevated in polysaccharides supplemented groups when compared to DHF group. GPx data also indicates the importance of percentage of polysaccharides in *P. tuber-regium*, because restored GPx was better in 1P than 2P and 3P (Figure 4).

## 4. Discussion

For the first time, medicinal importance of the polysaccharides extracted from edible mushroom *P. tuber-regium* was experimentally demonstrated against the adverse effects of diabetes. Three strains of polysaccharides exhibited potent antihyperglycemic, antihyperlipidemic, and antioxidant efficacies. This was revealed by decreased diabetic risk factors (blood glucose and HbA1c), lipid profiles (TC, TG, and LDL), attenuated lipid peroxidation (MDA), and restored antioxidant capacity (SOD and GPx). The therapeutic applications of polysaccharides appear to be associated with percentage of polysaccharides presence in each strain. 

Most experimental diabetic models were induced by employing a higher dose of STZ with a single or short-term injections [10, 13, 15]. However, higher doses of STZ ultimately destroyed pancreatic *β*-cells by necrosis that lead to severe changes in physiology and even death [22]. In our diabetic rat model, we used chronic lower doses of STZ combined with a high-fat diet to achieve diabetes that mimics the progression of human type 2 diabetes. We observed neither death rate nor severe adverse symptoms. Blood glucose levels increased after 4 weeks of injection that continued until 8 weeks. We assumed that STZ injection along with high-fat diet together caused negative physiological changes in the body, as evidenced by increased lipid profiles and decreased antioxidant status.

In our study, polysaccharides showed beneficial effects on maintaining bodyweights in diabetic rats fed a high-fat diet. This was clearly shown by lower bodyweights after 8-week treatment. Chronic high-fat diet consumption throughout this study resulted in an gradual increase of bodyweight. This increase lessened from the 2nd week after polysaccharide administration, which suggests that *P. tuber-regium* exerts weight management effects. Polysaccharides as nondigestible fibers may delay gastric emptying and influence nutrients (fat) absorption, which may results in lower weight gain [23]. 

Another major finding is that polysaccharide possesses potent antihyperglycemic effect as shown by decreased blood glucose levels in diabetic rats. Decreased blood glucose levels are corresponding to the decreased HbA1c in polysaccharides-treated groups. It has been shown that water soluble polysaccharides extracted from the sclerotia of *Inonotus obliquus* acts as a hypoglycemic substance [24]. Furthermore, *β*-glucans, a major bioactive ingredients in polysaccharides of many medicinal mushrooms may be responsible for lowering the blood glucose levels [25]. On the other hand, protection of pancreatic *β*-cells by polysaccharides [4] may attribute to its antidiabetic activity. 

STZ may harm the islets of Langerhans, thus causing the diabetic syndrome. Since hypoglycemic property of any substance mediated through stimulating insulin synthesis and/or secretion [26], increased insulin levels in our study indicate the capability of polysaccharides to modulate insulin secretion from *β*-cells. Wong and colleagues [4] speculates that polysaccharides of *P. citrinopileatus* may protect pancreatic *β*-cells or delay their impairment against STZ. Decreased insulin levels and pancreatic *β*-cell number in diabetic mice has been restored by treatment with *Inonotus obliquus* culture broth [25]. Moreover, chemical substances with antioxidant properties may help to regenerate *β*-cells and protect pancreatic islets against STZ toxicity [25]. Increased antioxidant capacity with polysaccharides perhaps plays an important role in decreasing *β*-cell damage, thereby restoring insulin levels. 

The reduction in lipid profile including TC, TG, and LDL in polysaccharide-treated diabetic rats indicates its antihyperlipidemic property. Drop in blood glucose levels by polysaccharides may be accompanied by decreased lipid profiles and increased HDL content. In general, hyperglycemic substances may also improve the metabolism of blood lipids. Jeong et al. [10] reported substantially decreased plasma TC and LDL levels that resulted from increased HDL levels by *Agaricus bisporus* powder in hypercholesterolemic rats. Administration of dry matter culture broth of *Inonotus obliquus* to diabetic mice significantly decreased the serum TC, TG, and LDL, while increased the HDL levels along with reversed tissue damages [25]. These results showed that mushroom extracts possess cholesterol lowering effect, which might be due to the presence of high fiber. According to one proposal, mushroom dietary fiber that contains polysaccharides might bind bile acids thereby reducing their entry into gut bile acid secretion [27]. Thus, liver responds by increasing hepatic conversion of cholesterol to bile acids that, therefore, results in reducing circulating cholesterol levels [28]. 

Hyperglycemia can trigger ROS production and promote glycation [29, 30], thereby increasing lipid peroxidation. Another key finding of our study showed hepatoprotective properties of polysaccharides by lowering the MDA levels against hyperglycemia. Sun et al. [25] reported decreased MDA content and increased antioxidant status with culture broth of *Inonotus obliquus* in the liver of diabetic mice. Antihyperglycemic property of polysaccharides in part may reduce the ROS production, therefore, causing decrease in MDA content. Besides, increased antioxidant status may also be a possible mechanism that explains reduced MDA levels. Moreover, polysaccharides play a vital role as free radical scavengers and protect tissues against oxidative damage [31].

SOD scavenges the superoxide radicals (O_2_^•−^) into hydrogen peroxide (H_2_O_2_), then GPx converts H_2_O_2_ into less toxic water and oxygen [32]. The increased production O_2_^•−^ either by STZ injections or high-fat diet results in SOD reduction [22]. Restored SOD (1P and 2P polysaccharides) and GPx (all polysaccharides) activities indicate effective elimination of ROS from diabetic liver, since diabetic rat represents elevated ROS levels and lower antioxidant capacity [22, 33]. It is well documented that polysaccharides of various mushrooms can restore the hepatic antioxidant status and decrease blood glucose levels in diabetic rats [10, 25]. Thus, high-percentage polysaccharides appear to be more effective to boost antioxidant status, thereby protects liver cells against hyperglycemia-induced oxidative damage.

## 5. Conclusions

For the first time, our findings demonstrated the antihyperglycemic, antihyperlipidemic, and antioxidant properties of *P. tuber-regium* polysaccharides in the STZ plus high-fat diet-induced diabetic rat model. Potent antihyperglycemic property of polysaccharides may attribute to reducing the lipid profile and oxidative stress. These therapeutic actions appear to be corresponding to the amount of polysaccharides present in each strain (1P > 2P > 3P). On the other hand, culture media of *P. tuber-regium* is low cost with high-percentage polysaccharides. Therefore, our study emphasizes the practical application of polysaccharides as an external supplement to cope diabetes plus high-fat diet-induced adverse effects. Our findings in the integrative medicine field provided a potential track to use *P. tuber-regium* polysaccharides as antihyperglycemic, antilipidemic, and antioxidant substances. 

## Figures and Tables

**Table 1:** The polysaccharide content in three different strains (1P, 2P, and 3P) of *Pleurotus tuber-regium*.

Strains	Polysaccharide content (%)
MUCL-39359 (1P)	8.18
MUCL-44597 (2P)	6.24
MUCL-44822 (3P)	3.99

The content of the polysaccharides was expressed in percentage (%) per total dry weight of each strain of *P. tuber-regium*.

**Table 2:** Effect of three different polysaccharides (1P, 2P, and 3P) on food intake and food conversion efficiency (FCE) in diabetic rats fed a high-fat diet (DHF).

Groups	Average food intake (g/day)	FCE ratio
DHF	28.28 ± 1.31	0.14 ± 0.13
DHF+1P	26.44 ± 1.66	0.17 ± 0.11
DHF+2P	28.11 ± 1.06	0.15 ± 0.12
DHF+3P	27.71 ± 1.13	0.15 ± 0.14

Values are expressed as mean ± SEM (*n* = 10). The food conversion efficiency (FCE) ratio = food intake (g)/weight gain (kg) × 10^2^.

**Table 3:** Changes in bodyweight and weight gain (g) over a period of 8 weeks in three different polysaccharides (1P, 2P, and 3P) supplemented and diabetic plus high-fat diet-fed rats (DHF).

	DHF	DHF+1P	DHF+2P	DHF+3P
Week 0	302.12 ± 2.1	290.82 ± 3.41	295.63 ± 3.1	292.35 ± 2.9
Week 2	360.29 ± 3.1	339.19 ± 2.76*	342.56 ± 3.1*	343.37 ± 2.97*
Week 4	430.71 ± 3.4	405.84 ± 3.67*	408.43 ± 2.66*	410.38 ± 3.2*
Week 6	469.28 ± 3.2	425.12 ± 3.54*	428.47 ± 3.15*	442.24 ± 2.99*
Week 8	499.8 ± 2.9	451.13 ± 3*	456.53 ± 3.33*	460.87 ± 3.12*

Weight gain	197.7 ± 4	160.31 ± 4.1*	190.09 ± 3.54^†^	188.52 ± 3^†#^

Values are expressed as mean ± SEM (*n* = 10). The ∗ indicates a significant difference compared to DHF group at respective week (*P* < 0.01), † indicates a significant compared DHF+1P (*P* < 0.01), and # indicates a significant difference compared to DHF+2P (*P* < 0.05).

**Table 4:** Changes in blood glucose, insulin, and HbA1c levels in diabetic rats fed a high-fat (DHF) and treated with three different polysaccharides (1P, 2P, and 3P).

	DHF	DHF+1P	DHF+2P	DHF+3P
Glucose (mg/dL)	368.0 ± 2.3	270.6 ± 2.5*	288.4 ± 1.7^∗†^	311.1 ± 1.3^∗†^
Insulin (*μ*U/mL)	0.3 ± 0.1	0.9 ± 0.1*	0.8 ± 0.1*	0.6 ± 0.1^∗†^
HbA1c (%)	9.4 ± 0.5	5.6 ± 0.4*	5.9 ± 0.3*	6.3 ± 0.4^∗†^

Values are expressed as mean ± SEM (*n* = 10). The ∗ indicates a significant difference compared to DHF group (*P* < 0.01) and † indicates a significant difference compared to DHF+1P (*P* < 0.01).

**Table 5:** Effect of three different polysaccharides (1P, 2P, and 3P) on total cholesterol (TC), triglyceride (TG), HDL, and LDL levels in diabetic rats fed a high-fat diet (DHF).

	DHF	DHF+1P	DHF+2P	DHF+3P
TC (mg/dL)	155.7 ± 2.5	119.5 ± 2.6*	131.1 ± 3.1^∗†^	137.6 ± 2.1^∗†#^
TG (mg/dL)	192.3 ± 2.4	178.9 ± 2.8*	181.7 ± 2.5*	183 ± 2.4*
HDL (mg/dL)	26.1 ± 2.5	33 ± 1.8*	30.7 ± 2.3*	29.3 ± 1.3*
LDL (mg/dL)	191.1 ± 3.0	151 ± 1.6*	164 ± 1.5^∗†^	171.7 ± 2.4^∗†#^

Values are expressed as mean ± SEM (*n* = 10). The ∗ indicates a significant difference compared to DHF group (*P* < 0.05), † indicates a significant difference compared to DHF+1P (*P* < 0.01), and # indicates a significant difference compared to DHF+2P (*P* < 0.01).
